# Pacemaker Implantation in Patients With Pre-Existing Bundle Branch Block Undergoing Balloon-Expandable Transcatheter Aortic Valve Replacement

**DOI:** 10.1016/j.shj.2026.101052

**Published:** 2026-05-22

**Authors:** Judah Rajendran, Shivabalan Kathavarayan Ramu, Carl Ammoury, Besir Besir, Akhilesh Khuttan, Odette Iskander, Tamari Lomaia, Ankit Agrawal, Alexander Egoavil, James Yun, Grant Reed, Rishi Puri, Serge Harb, Oussama Wazni, Amar Krishnaswamy, Samir R. Kapadia

**Affiliations:** Department of Cardiovascular Medicine, Heart, Vascular and Thoracic Institute, Cleveland Clinic, Cleveland, Ohio

**Keywords:** Atrial fibrillation/flutter, Implantation depth, Left bundle branch block (LBBB), Permanent pacemaker (PPM), Right bundle branch block (RBBB), TAVR

## Abstract

**Background:**

The primary objective of this study was to compare permanent pacemaker (PPM) implantation rates following balloon-expandable transcatheter aortic valve replacement (TAVR) between patients with pre-existing right bundle branch block (RBBB) versus left bundle branch block (LBBB). Secondary objectives were to assess long-term clinical outcomes according to baseline conduction status and post-TAVR PPM implantation and identify independent predictors of PPM implantation.

**Methods:**

This retrospective study included 380 patients with pre-existing RBBB (n = 269) and LBBB (n = 111) who underwent balloon-expandable TAVR between 2016 and 2020, excluding those with prior pacemakers or implantable cardioverter defibrillators.

**Results:**

Within 30 days following TAVR, PPM implantation was significantly more frequent in patients with preexisting RBBB compared with LBBB (18.59 vs. 7.21%, *p* = 0.003), whereas early mortality remained low and similar between groups. Patients with RBBB exhibited larger annular dimensions, greater annular calcium burden, and a narrower membranous septum angle. Over long-term 2-year follow-up, there were no significant differences in all-cause mortality or heart failure hospitalization between RBBB and LBBB patients, irrespective of post-TAVR PPM implantation status. On multivariable logistic regression, baseline RBBB (odds ratio [OR] 3.56; 95% CI 1.01–12.58) and prior atrial fibrillation/flutter (OR 4.13; 95% CI 1.63–10.45) were independently associated with increased 30-day PPM risk, whereas deeper right-sided membranous septum implantation depth was associated with lower PPM risk (OR 0.90; 95% CI 0.81–0.99).

**Conclusions:**

Patients with preexisting RBBB undergoing balloon-expandable TAVR had a significantly higher and earlier rate of PPM implantation compared to LBBB patients, with no differences in outcomes irrespective of BBB and PPM implantation status. Atrial fibrillation/flutter and RBBB were associated with an increased risk of 30-day PPM implantation, although the risk is acceptable with higher valve implantation.

## Highlights

• Permanent pacemaker (PPM) implantation was earlier and more frequent in transcatheter aortic valve replacement (TAVR) patients with right bundle branch block (RBBB) vs. left bundle branch block (LBBB).
• There were no differences in mortality or HF hospitalization between RBBB and LBBB.
• Pre-TAVR RBBB and atrial fibrillation (AF) increased 30-day PPM risk, while deeper septal depth lowered risk.
• Complete heart block (CHB) was the main PPM indication in RBBB, while sudden cardiac death prevention was more common in LBBB.

## Introduction

Transcatheter aortic valve replacement (TAVR) is now the preferred minimally invasive treatment for severe aortic stenosis (AS), particularly for patients deemed high risk for surgical aortic valve replacement.^1^ However, a notable limitation is the increased risk of permanent pacemaker (PPM) implantation due to conduction disturbances, as the proximity of the aortic valve to the His-Purkinje conduction system makes conduction injury more likely than with surgical aortic valve replacement.^2^^,^^3^ Although studies and meta-analyses mostly report a gradual decline in PPM rates after TAVR, site-level variability persists.^4^^,^^5^

Patients with preexisting conduction abnormalities, particularly right bundle branch block (BBB) (RBBB), are at significantly higher risk of requiring PPM after TAVR.^6^ A recent meta-analysis reported a 30-day PPM rate of 38.1% in patients with RBBB compared to 11.4% in those without, yielding a relative risk (RR) of 3.56.^6^ Similarly, preexisting left BBB (LBBB), present in 9%-12% of patients, is linked to increased PPM rates within 30 days after TAVR.^7^

Despite these known risks, data specific to patients with baseline BBB remain limited.^7^, ^8^, ^9^ This study aimed to identify patient-specific and procedural predictors of PPM and compare post-TAVR outcomes between patients with preexisting RBBB and LBBB.

## Methods

### Study Population

**Figure 1:**
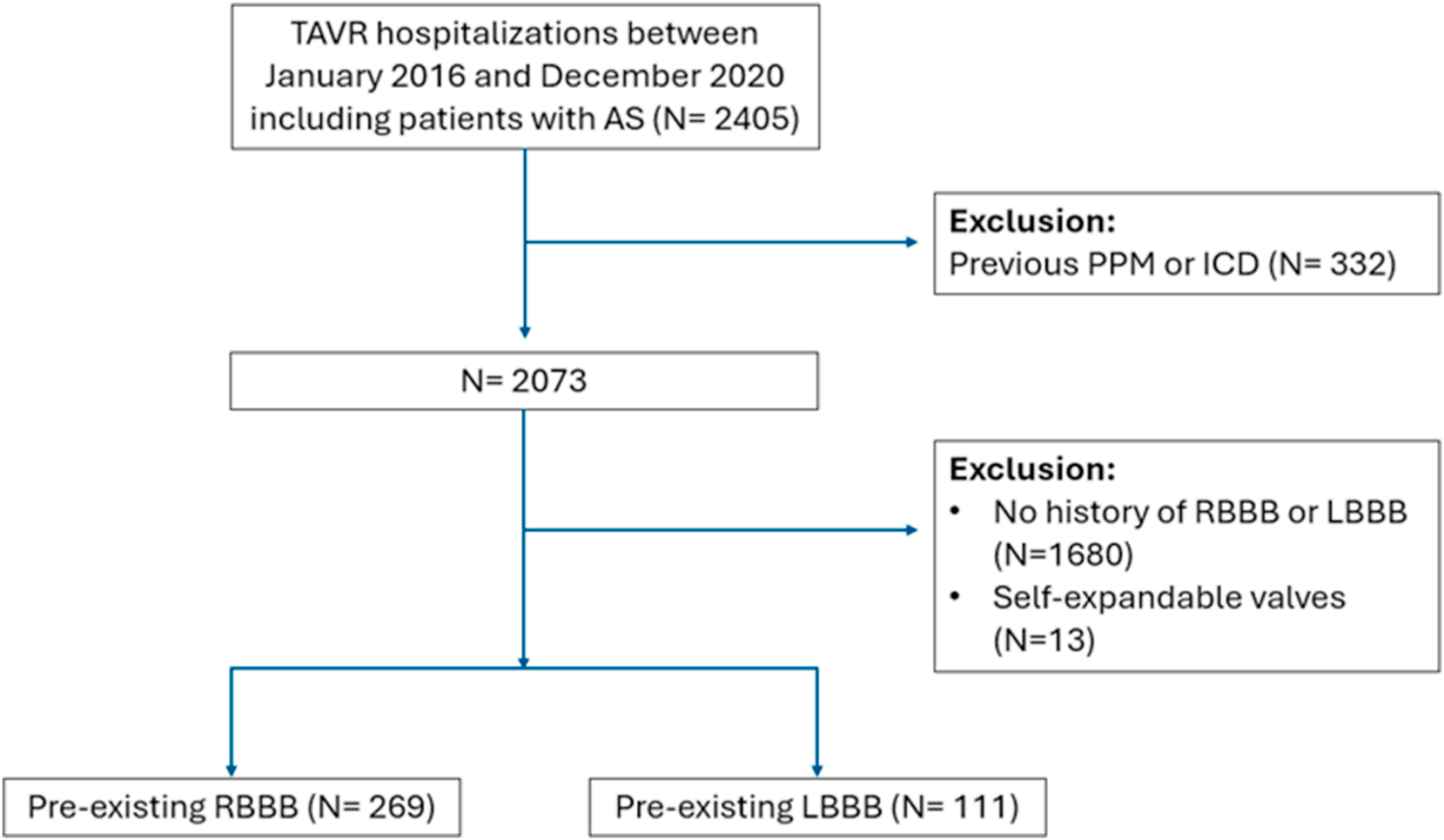
Study flow. Abbreviations: AS, aortic stenosis; ICD, implantable cardioverter defibrillator; LBBB, left bundle branch block; PPM, permanent pacemaker; RBBB, right bundle branch block; TAVR, transcatheter aortic valve replacement.

This retrospective study involved patients with AS who underwent TAVR between January 1, 2016, and December 31, 2020, at our institution. The study included patients with preexisting BBB, divided into 2 groups based on RBBB or LBBB at baseline (see Figure 1). Patients who had a preexisting PPM or implantable cardioverter defibrillator (ICD) before the procedure were excluded. Patients who underwent TAVR with self-expandable valve platforms were also excluded. The study received approval from the institutional review board (IRB 24-070).

### Echocardiographic Evaluation

The study included a comprehensive echocardiographic evaluation for all patients, conducted using widely accessible standard ultrasonography systems. Skilled echocardiographers, blinded to the patients’ clinical information, meticulously examined and measured all echocardiographic parameters in accordance with established guidelines of echocardiographic evaluation of stenotic lesion.^10^ Echocardiograms performed within 1 year before the TAVR procedure were considered baseline readings. Follow-up echocardiograms were conducted immediately after TAVR before discharge and at 30 days, 1 year, and 2 years postprocedure. Key parameters assessed included the mean and peak transvalvular aortic gradient, dimensionless valve index, left ventricular ejection fraction, and left ventricular end-diastolic and systolic dimensions and volumes.

### Electrocardiographic Evaluation

Twelve-lead electrocardiograms (ECGs) were performed at a minimum of 3 time points: before TAVR, immediately after TAVR, and on day 0 after TAVR. All ECG findings were evaluated based on standard definitions and guidelines set forth by the American Heart Association, the American College of Cardiology Foundation, and the Heart Rhythm Society.^11^ RBBB was defined as a QRS duration >120 ms with typical RBBB morphology (rR′ in V1).^11^ LBBB was defined as a QRS duration >120 ms with a QRS complex that is negative in V1, showing a small R wave or no R wave.^11^ Bifascicular block was defined as the combination of RBBB with either left anterior fascicular block (LAFB) or left posterior fascicular block. Procedural high-grade atrioventricular block (HAVB)/complete heart block (CHB) was defined as any HAVB/CHB episode occurring during the transcatheter aortic valve implantation procedure; delayed HAVB/CHB was defined as any HAVB/CHB episode occurring after the patient had left the procedure room and within 30 days after the procedure. Telemetry is continued until discharge. An ECG patch (Zio Patch; iRhythm, San Francisco, California) is used for ambulatory ECG monitoring following discharge at the discretion of operating physicians.

### Multidimensional Computed Tomography Evaluation

All patients underwent a pre-TAVR multidimensional computed tomography (CT) scan, providing comprehensive heart coverage, retrospective ECG gating, and multiphase reconstructions of the aortic root throughout the cardiac cycle. This approach enabled dynamic 4-dimensional rendering for valve function evaluation. To optimize safety and image quality, radiation and contrast doses were adjusted individually for each patient, and high spatial resolution was used. Computed tomographic images were analyzed by independent readers using dedicated software (Aquarius iNtuition v4.4; TeraRecon, Foster City, California), facilitating a detailed assessment of aortic valve and individual leaflet calcification in accordance with established diagnostic guidelines. Measurements of the virtual aortic basal ring were obtained from double-oblique images derived from standard coronal, sagittal, and transverse views, as recommended by Achenbach et al.^12^ For accuracy, the imaging plane was positioned at the level of the basal leaflet attachments, with adjustments to the transverse plane to ensure simultaneous visualization of the leaflet nadirs.

Calcium deposits within the basal ventricular septum were evaluated in the left ventricular outflow tract, with the software providing both calcium volume and scoring data via a specialized module.^13^ Membranous septal length (MSL) was also measured in this view by first focusing on the thinnest portion of the interventricular septum. The vertical distance from the visible tip of the muscular septum to the annular plane was then measured using the corresponding stretched vessel image, ensuring an accurate assessment. The eccentricity index (EI) measures how much the shape of the aortic annulus (AA) differs from a perfect circle. It was calculated as EI = 1 − (minimal diameter/maximum diameter). An EI of 0 represents a perfect circle, whereas values above 0.1 indicate a more oval shape. Although originally developed to predict paravalvular leak after TAVR, it has been recently shown to predict new PPM, as well.^14^^,^^15^ We also measured the oversizing index of the AA area. It was calculated as ([nominal valve area − measured area]/nominal valve area) × 100. Again, it was originally developed to optimize reduction of paravalvular leak severity, but excessive oversizing has been shown to increase PPM rates.^16^^,^^17^ The angle between the plane of the AA and membranous septum (MS) (AA-MS angle) was also measured as previously described to be usually oblique in patients requiring PPM.^18^

### Fluoroscopic Evaluation

Implantation depth (ID) was determined using fluoroscopy images in both the right anterior oblique (RAO) and left anterior oblique (LAO) projections. ID was measured from the tip of the cusp (on both the noncoronary/right cusp side and the left coronary cusp side) to the most proximal edge of the implanted valve in the final angiogram after valve deployment using the SyngoDynamics software (Malvern, Pennsylvania). In addition, the Δ membranous septum ID (MSID) was calculated by measuring the difference between the MSL obtained via CT and the ID.^19^ MSID was calculated separately for the RAO and LAO projections on either side. MSID on the right side was calculated as the average of the MSID on the non-coronary cusp (NCC)/right coronary cusp (RCC) side for the RAO and LAO projections, whereas MSID on the left side was calculated as the average of the MSID on the LCC side for the RAO and LAO projections. The mean MSID was obtained as the average of the MSID on the right and left sides.

### Statistical Analysis

Categorical variables were analyzed using either the Chi-square test or Fisher's exact test, with results presented as numbers and percentages. The independent t-test was applied to analyze continuous variables, reported as mean ± SD. A multivariate logistic regression model incorporating baseline clinical, anatomical, and procedural factors was developed to identify factors predictive of PPM after TAVR. In logistic regression analyses, the primary endpoint was early PPM occurring within 30 days following TAVR. Secondary endpoints included cumulative PPM implantation during the entire available follow-up period, extending up to 2 years after the index procedure. A multivariate linear regression model was developed and validated to assess if in-hospital pacemaker placement was associated with prolonged hospital stay or not. For linear regression analyses, estimates represent standardized β coefficients, reflecting the average change in length of stay (in days) per 1 SD increase in the predictor variable or relative to the reference category for categorical variables. Predictor variables included in multivariate models were selected a priori based on clinical relevance and biological plausibility. Kaplan–Meier analyses were conducted. A 2-tailed *p*-value of <0.05 is reported to be statistically significant. Statistical analysis was conducted using SPSS Software (version 23.0; IBM Corporation, Armonk, New York) and R Software (version 4.2.2; R Project for Statistical Computing, Vienna, Austria).

## Results

The study population included 380 patients with preexisting BBB, of which 269 patients (70.80%) had pre-existing RBBB and 111 patients (29.20%) had preexisting LBBB (Figure 1). The average age was similar between groups (RBBB: 79.88 ± 8.71 years, LBBB: 81.03 ± 7.55 years). Female representation was higher in the LBBB group (52.25 vs. 28.99%, *p*-value <0.001). Diabetes mellitus, chronic obstructive pulmonary disease, and prior transient ischemic attacks were significantly more prevalent in RBBB patients (Table 1). LBBB patients had a lower left ventricular ejection fraction (49.00% ± 13.70% vs. 57.11% ± 10.22%, *p*-value <0.001), larger LV end-systolic diameter (3.44 ± 0.89 cm vs. 3.21 ± 0.78 cm, *p*-value = 0.02), and smaller AV area (0.71 ± 0.19 cm^2^ vs. 0.78 ± 0.21 cm^2^, *p*-value = 0.01). In addition, a greater proportion of patients in the LBBB group presented with severe left ventricular hypertrophy (30.63% vs. 18.59%, *p*-value = 0.01) and a more frequent presence of moderate or greater mitral regurgitation (≥ 2+: 22.52% vs. 13.38%, *p*-value = 0.04) (Table 1). LAFB was present in 25.28% of the RBBB group. Baseline multidimensional CT characteristics showed larger annular dimensions in the RBBB group. The RBBB group had significantly higher calcium scores across all aortic cusps and the left ventricular outflow tract (Table 1).

**Table 1:** Baseline characteristics

Baseline characteristic	Summary statistics, n = 380 (100%)	Pre-TAVR RBBB, n = 269 (70.80%)	Pre-TAVR LBBB, n = 111 (29.20%)	*p*-value
Clinical characteristics				
Age, y	80.21 ± 8.39	79.88 ± 8.71	81.03 ± 7.55	0.23
Female sex, n (%)	136 (35.79%)	78 (28.99%)	58 (52.25%)	**<0.001**
STS risk score				
<4%, n (%)	132 (34.74%)	103 (38.29%)	29 (26.13%)	**0.02**
4%–8%, n (%)	153 (40.26%)	100 (37.17%)	53 (47.75%)	0.06
>8%, n (%)	95 (25.00%)	66 (24.53%)	29 (26.13%)	0.75
Body mass index, m^2^	29.55 ± 6.70	29.79 ± 6.73	28.96 ± 6.61	0.27
Body surface area, kg/m^2^	1.98 ± 0.28	1.98 ± 0.29	1.96 ± 0.27	0.43
History of syncope, n (%)	15 (3.95%)	14 (5.20%)	1 (0.90%)	0.08
NYHA III or IV, n (%)	294 (77.37%)	209 (77.70%)	85 (76.58%)	0.81
Coronary artery disease, n (%)	214 (56.31%)	157 (58.36%)	57 (51.35%)	0.21
Hypertension, n (%)	344 (90.52%)	247 (91.82%)	97 (87.39%)	0.18
Diabetes mellitus, n (%)	158 (41.58%)	122 (45.35%)	36 (32.43%)	**0.02**
Current dialysis, n (%)	14 (3.68%)	13 (4.83%)	1 (0.90%)	0.08
History atrial fibrillation or flutter, n (%)	141 (37.11%)	99 (36.80%)	42 (37.83%)	0.85
Peripheral artery disease, n (%)	26 (6.84%)	17 (6.32%)	9 (8.11%)	0.53
Chronic obstructive lung disease, n (%)	61 (16.05%)	51 (18.96%)	10 (9.01%)	**0.02**
Native bicuspid aortic valve, n (%)	26 (6.84%)	18 (6.69%)	8 (7.21%)	0.86
Prior stroke, n (%)	53 (13.95%)	37 (13.75%)	16 (14.41%)	0.87
Prior transient ischemic attack, n (%)	39 (10.26%)	33 (12.27%)	6 (5.40%)	**0.04**
Prior PCI, n (%)	112 (29.47%)	86 (31.97%)	26 (23.42%)	0.10
Prior CABG, n (%)	119 (31.32%)	92 (34.20%)	27 (24.32%)	0.06
Echocardiographic characteristics				
Days from ECHO to TAVR, median IQR	119.00 (68.00-194.00)	120.50 (69.00-199.5)	110.50 (65.50-189.8)	0.77
LVEF, %	54.69 ± 11.94	57.11 ± 10.22	49.00 ± 13.70	**<0.001**
AV area, cm^2^	0.76 ± 0.20	0.78 ± 0.21	0.71 ± 0.19	**0.01**
AV area index, cm^2^/m^2^	0.39 ± 0.11	0.39 ± 0.11	0.38 ± 0.10	0.23
AV mean gradient, mmHg	41.06 ± 14.10	41.53 ± 14.34	39.94 ± 13.51	0.33
LV end diastolic diameter, cm	4.69 ± 0.85	4.69 ± 0.84	4.70 ± 0.89	0.89
LV end systolic diameter, cm	3.28 ± 0.82	3.21 ± 0.78	3.44 ± 0.89	**0.02**
LA volume index biplane, ml/m^2^	45.84 ± 16.96	45.17 ± 16.64	47.47 ± 17.70	0.26
Left ventricular mass index, g/m^2^	115.99 ± 37.57	114.73 ± 39.60	118.95 ± 32.33	0.34
AR degree ≥2+	40 (10.53%)	28 (10.41%)	12 (10.81%)	0.96
MR degree ≥2+	61 (16.05%)	36 (13.38%)	25 (22.52%)	**0.04**
LVH severity				
Mild, n (%)	59 (15.53%)	41 (15.24%)	18 (16.22%)	0.99
Moderate, n (%)	38 (10.00%)	29 (10.78%)	9 (8.11%)	0.39
Severe, n (%)	84 (22.11%)	50 (18.59%)	34 (30.63%)	**0.01**
Baseline ECG characteristics				
Ventricular rate, bpm	72.27 ± 13.99	72.72 ± 14.14	71.17 ± 13.65	0.33
PR interval, ms	192.98 ± 41.65	194.37 ± 40.43	189.70 ± 44.44	0.35
QRS duration, ms	147.06 ± 15.62	146.52 ± 15.44	148.36 ± 16.04	0.30
First-degree AV block, n (%)	120 (31.58%)	85 (31.60%)	35 (31.53%)	0.99
Left anterior fascicular block, n (%)	68 (17.89%)	68 (25.28%)	0 (0.00%)	**<0.001**
Left posterior fascicular block, n (%)	2 (0.53%)	2 (0.74%)	0 (0.00%)	1.00
MDCT characteristics				
Annular area, mm^2^	484.50 ± 91.23	494.30 ± 91.52	460.55 ± 86.33	**0.001**
Perimeter, mm	80.09 ± 7.73	80.73 ± 7.72	78.39 ± 7.52	**0.01**
Eccentricity index	0.19 ± 0.07	0.19 ± 0.07	0.20 ± 0.07	0.22
MSL, mm	12.33 ± 4.40	12.57 ± 4.39	11.68 ± 4.40	0.13
MS-AA angle	94.53 ± 8.32	93.23 ± 7.29	97.79 ± 9.77	**<0.001**
NCC calcium score, AU	445.01 ± 367.36	472.90 ± 373.41	373.26 ± 342.74	0.02
NCC calcium volume, cu.mm	338.19 ± 279.35	360.92 ± 284.93	279.73 ± 256.62	**0.01**
RCC calcium score, AU	348.16 ± 330.08	388.19 ± 333.43	245.18 ± 298.97	**<0.001**
RCC calcium volume, cu.mm	265.23 ± 249.92	297.02 ± 255.55	183.44 ± 215.22	**<0.001**
LCC calcium score, AU	359.62 ± 353.93	385.64 ± 366.06	292.69 ± 312.42	**0.02**
LCC calcium volume, cu.mm	270.13 ± 267.15	290.44 ± 279.30	217.90 ± 225.96	**0.01**
Total calcium score, AU	1149.79 ± 933.08	1239.81 ± 936.41	918.17 ± 887.62	**0.002**
Total calcium volume, cu.mm	871.19 ± 707.14	944.81 ± 716.80	681.07 ± 646.99	**0.001**
LVOT calcium score, AU	300.79 ± 615.67	319.34 ± 696.79	98.68 ± 223.45	**<0.001**
LVOT calcium volume, cu.mm	234.34 ± 471.70	296.80 ± 532.70	72.05 ± 166.45	**<0.001**

*Notes*. Significant *p*-values (<0.05) are highlighted in bold.

Abbreviations: AA, aortic annulus; AR, aortic regurgitation; AU, agatston unit; AV, atrioventricular; bpm, beats per minute; CABG, coronary artery bypass graft; ECG, electrocardiogram; ECHO, echocardiography; IQR, interquartile range; LA, left atrial; LBBB, left bundle branch block; LCC, left coronary cusp; LV, left ventricular; LVEF, left ventricular ejection fraction; LVH, left ventricular hypertrophy; LVOT, left ventricular outflow tract; MDCT, multidimensional computed tomography; MR, mitral regurgitation; MS, membranous septum; MSL, membranous septal length; NCC, non-coronary cusp; NYHA, New York Heart Association; PCI, percutaneous coronary intervention; RBBB, right bundle branch block; RCC, right coronary cusp; STS, Society of Thoracic Surgeons; TAVR, transcatheter aortic valve replacement.

**Figure 2:**
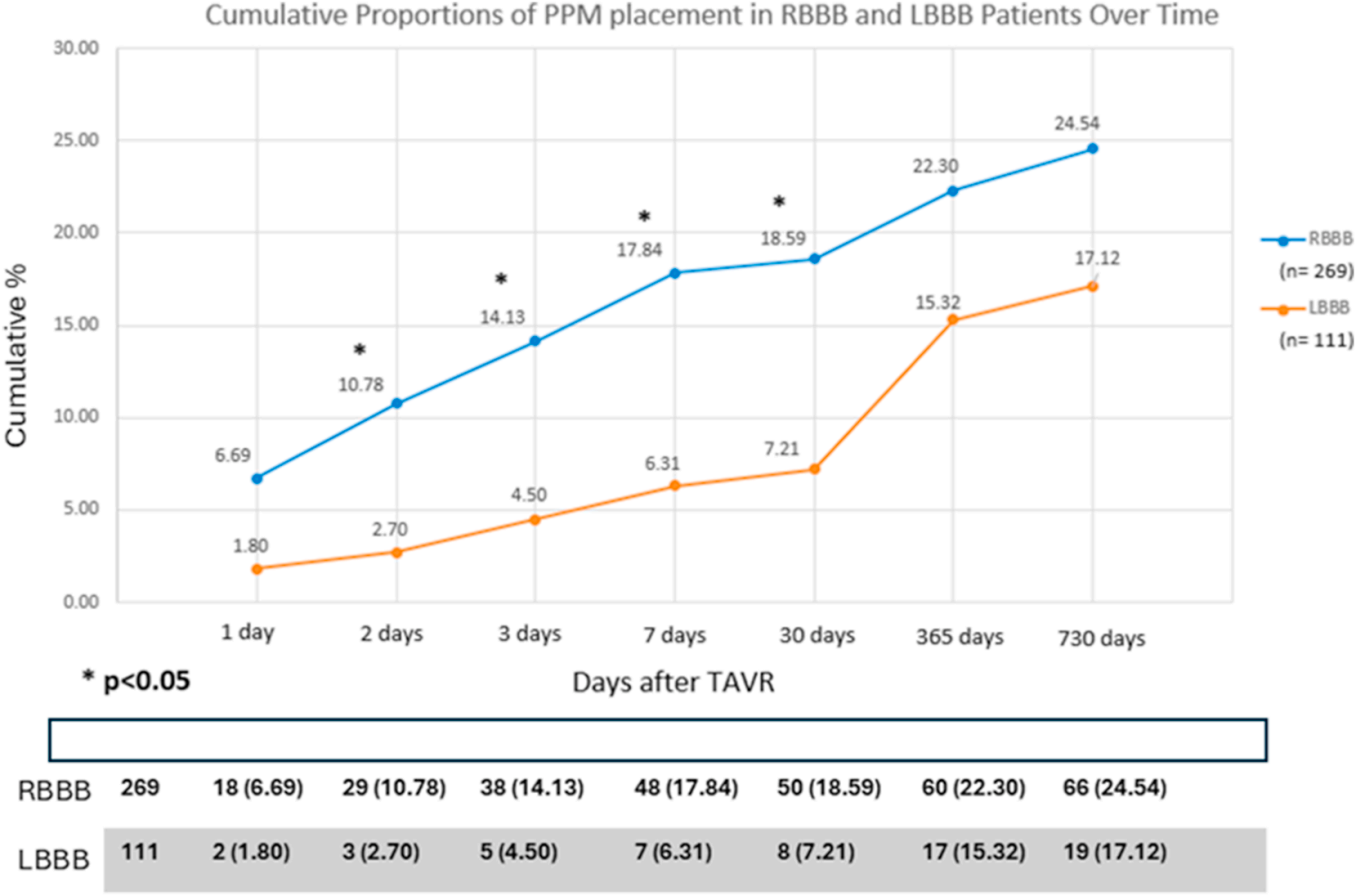
Number and proportion of permanent pacemaker placements by days after TAVR. Abbreviations: LBBB, left bundle branch block; PPM, permanent pacemaker; RBBB, right bundle branch block; TAVR, transcatheter aortic valve replacement.

Procedural CHB was significantly more frequent in the RBBB group (12.27% vs. 0.90%, *p*-value <0.001, Table 2). On day 0 post-TAVR, the RBBB group had a longer PR interval (222.10 ± 58.29 ms vs. 205.84 ± 47.93 ms, *p*-value = 0.02), and a greater PR interval increase after TAVR (*p*-value = 0.001). New LAFB occurred in 4.09% of the RBBB group. In-hospital, 30-day, and 2-year PPM implantation rates are reported as the proportion of patients with pre-TAVR LBBB or RBBB who required PPM placement during the respective timeframes. In-hospital PPM implantation was more frequent in the RBBB group (16.36% vs. 6.30%, *p*-value = 0.01). Among the 329 patients discharged without a permanent pacemaker, 13 (3.95%) were readmitted within 30 days due to conduction disturbances: 12 of 225 patients with pre-TAVR RBBB (5.33%) and 1 of 104 patients with pre-TAVR LBBB (0.96%). A total of 13 patients (3.95%) were readmitted within 30 days due to conduction disturbances, including 12 patients with pre-TAVR RBBB (4.46%) and 1 patient with pre-TAVR LBBB (0.90%). Early 30-day readmission due to conduction disturbances did not differ significantly between RBBB and LBBB groups (*p*-value = 0.07). Figure 2 shows the cumulative incidence of PPM implantation after TAVR in patients with preexisting RBBB and LBBB. PPM occurred more frequently and earlier in RBBB. At 1 day postprocedure, the difference between groups was not statistically significant (*p*-value = 0.1; RR, 3.72; 95% CI, 0.95–14.5), but from days 2 through 30, RBBB showed significantly higher PPM rates (*p*-value <0.05 for days 2, 3, 7, and 30) with RRs of 3.99 (95% CI, 1.25–12.8), 3.14 (95% CI, 1.27–7.70), 2.83 (95% CI, 1.32–6.05), and 2.54 (95% CI, 1.25–5.16), respectively. By day 7, 17.84% of RBBB patients vs. 6.31% of LBBB patients had received PPM. This difference persisted during long-term follow-up but was no longer statistically significant at 1 or 2 years (all *p*-values >0.05). Cumulative PPM rates reached 22.30% vs. 15.32% at 1 year and 24.54% vs. 17.12% at 2 years (RR = 1.46 and 1.43, respectively). These findings indicate that RBBB is associated with an earlier and higher incidence of PPM after TAVR, particularly within the first month.

**Table 2:** Procedural characteristics and outcomes

Procedural characteristic	Summary statistics (n = 380)	Pre-TAVR RBBB, n = 269	Pre-TAVR LBBB, n = 111	*p*-value
Nontransfemoral access site, n (%)	21 (5.52%)	14 (5.20%)	7 (6.30%)	0.67
Valve size				
20 mm, n (%)	16 (4.21%)	7 (2.60%)	9 (8.10%)	**0.02**
23 mm, n (%)	101 (26.58%)	65 (24.16%)	36 (32.43%)	0.08
26 mm, n (%)	143 (37.63%)	103 (38.29%)	40 (36.04%)	0.74
29 mm, n (%)	105 (27.63%)	84 (31.23%)	21 (18.92%)	**0.02**
Concomitant PCI, n (%)	6 (1.58%)	3 (1.12%)	3 (2.70%)	0.36
Predilation performed, n (%)	69 (18.2%)	49 (18.22%)	20 (18.02%)	0.96
Deployment volume				
Nominal minus volume	157 (41.31%)	113 (42.00%)	44 (36.63%)	0.69
Nominal volume	183 (48.16%)	127 (47.21)	56 (50.45%)	0.67
Nominal plus volume	40 (10.53%)	29 (10.78%)	11 (9.91%)	0.94
Postdilation balloon volume				
Postdilation performed, n (%)	145 (38.16%)	107 (39.78%)	38 (34.23%)	0.31
Nominal minus volume	34 (8.94%)	24 (8.92%)	10 (9.01%)	1.00
Nominal volume	30 (7.89%)	24 (8.92%)	6 (5.40%)	0.25
Nominal plus volume	81 (21.32%)	59 (21.93%)	22 (19.81%)	
ECG characteristics on day 0 post-TAVR				
Procedural complete heart block, n (%)	34 (8.94%)	33 (12.27%)	1 (0.90%)	**<0.001**
Ventricular rate, bpm	64.63 ± 13.82	65.36 ± 14.08	62.84 ± 13.06	0.11
PR interval, ms	216.88 ± 55.61	222.10 ± 58.29	205.84 ± 47.93	**0.02**
QRS duration, ms	149.58 ± 19.51	149.08 ± 19.64	150.79 ± 19.25	0.45
First-degree AV block, n (%)	151 (39.74%)	110 (40.89%)	41 (36.94%)	0.47
Left anterior fascicular block, n (%)	11 (2.89%)	11 (4.09%)	0 (0.00%)	**0.04**
Second degree AV block, n (%)	4 (1.05%)	4 (1.49%)	0 (0.00%)	0.33
Rhythm: sinus, n (%)	254 (66.84%)	174 (64.68%)	80 (72.07%)	0.09
Rhythm: atrial fibrillation, n (%)	33 (8.68)	20 (7.43%)	13 (11.71%)	0.26
Rhythm: atrial flutter, n (%)	4 (1.05%)	4 (1.49%)	0 (0.00%)	0.46
Rhythm: junctional, n (%)	8 (2.11%)	6 (2.23%)	2 (1.80%)	1.00
Delta ventricular rate	−7.79 ± 15.52	−7.57 ± 16.10	−8.32 ± 14.07	0.68
Delta PR interval	12.39 ± 22.35	14.82 ± 25.44	7.21 ± 12.25	**0.001**
Delta QRS duration	2.28 ± 11.15	2.32 ± 11.61	2.18 ± 9.98	0.91
Ziopatch at discharge, n (%)	29 (7.63%)	26 (9.67%)	3 (2.70%)	0.03
Valve implantation outcomes				
Oversizing index	8.80 ± 11.09	8.88 ± 11.59	8.60 ± 9.80	0.83
Implantation depth LAO RCC, mm	3.64 ± 2.60	3.52 ± 2.43	3.94 ± 2.95	0.21
Implantation depth LAO LCC, mm	3.77 ± 2.22	3.82 ± 2.34	3.67 ± 1.91	0.56
Implantation depth RAO NCC, mm	2.80 ± 2.11	2.94 ± 2.13	2.45 ± 2.03	0.05
Implantation depth RAO RCC, mm	4.29 ± 2.03	4.36 ± 2.07	4.10 ± 1.92	0.28
MSID right side, mm	9.30 ± 4.77	9.48 ± 4.85	8.82 ± 4.57	0.35
MSID left side, mm	8.37 ± 4.56	8.57 ± 4.66	7.83 ± 4.26	0.28
Mean MSID, mm	8.83 ± 4.59	9.02 ± 4.68	8.33 ± 4.32	0.30
Procedural complications and outcomes				
Overall length of stay, d	3.62 ± 5.08	3.60 ± 4.73	3.67 ± 5.88	0.91
Postprocedure length of stay, d	3.00 ± 3.36	2.99 ± 2.97	3.04 ± 4.19	0.92
In-hospital mortality, n (%)	0 (0.00%)	0 (0.00%)	0 (0.00%)	-
In hospital ischemic stroke, n (%)	4 (1.05%)	3 (1.12%)	1 (0.90%)	1.00
In-hospital PPM, n (%)	51 (13.42%)	44 (16.36%)	7 (6.30%)	**0.01**
Bleeding access site in hospital, n (%)	7 (1.84%)	6 (2.23%)	1 (0.90%)	0.68
Hematoma access in hospital, n (%)	7 (1.84%)	5 (1.86%)	2 (1.80%)	1.00
Other bleed in hospital, n (%)	4 (1.05%)	3 (1.11%)	1 (0.90%)	1.00
New dialysis in hospital, n (%)	2 (0.53%)	1 (0.37%)	1 (0.90%)	0.50
PPM implantation within 30 d, n (%)	58 (15.26%)	50 (18.59%)	8 (7.21%)	**0.01**
PPM implantation within 2 y, n (%)	85 (22.37%)	66 (24.54%)	19 (17.12%)	0.12

*Notes*. Significant *p*-values (<0.05) are highlighted in bold.

Abbreviations: AV, atrioventricular; ECG, electrocardiogram; LAO, left anterior oblique; LBBB, left bundle branch block; LCC, left coronary cusp; MS, membranous septum; MSID, membranous septum implantation depth; NCC, non-coronary cusp; PCI, percutaneous coronary intervention; RCC, right coronary cusp; PPM, permanent pacemaker; RAO, right anterior oblique; RBBB, right bundle branch block; TAVR, transcatheter aortic valve replacement.

## Clinical Outcomes

**Figure 3:**
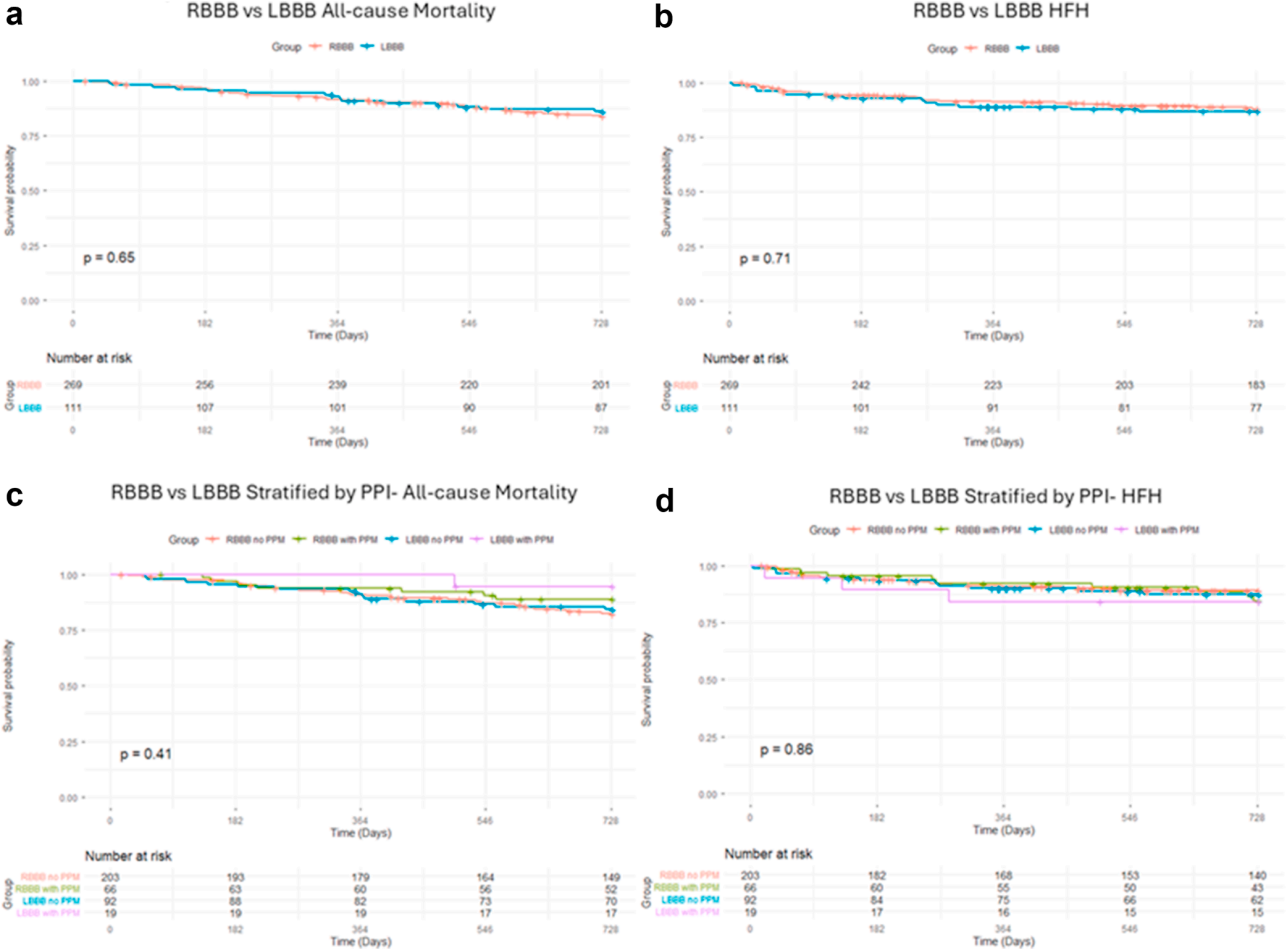
Kaplan–Meier survival curves for all-cause mortality and heart failure hospitalization (HFH) at 2 years. Abbreviations: HFH, heart failure hospitalization; LBBB, left bundle branch block; PPI, permanent pacemaker implantation; RBBB, right bundle branch block.

At 1 year, 89.48% of patients remained in active follow-up, with 2.63% lost to follow-up and 7.89% deceased. By 2 years, follow-up retention declined to 75.52%, with 9.74% lost and 14.74% deceased. Kaplan–Meier analysis showed no significant differences in all-cause mortality (*p*-value = 0.65) or heart failure hospitalization (*p*-value = 0.71) between RBBB and LBBB groups at 2 years (Figure 3a and b). Stratified analysis by PPM status yielded consistent findings, with no differences in all-cause mortality (*p*-value = 0.41) or heart failure hospitalization (*p*-value = 0.86) across subgroups (Figure 3c and d). Although RBBB patients experienced higher and earlier PPM implantation rates as shown in Figure 2, our results indicate that the need for pacing did not translate into adverse clinical outcomes, underscoring that overall post-TAVR prognosis remained favorable regardless of baseline conduction pattern or PPM status.

### Pacemaker Indications

CHB was the most common indication for PPM, significantly more frequent in the RBBB group and predominantly requiring in-hospital implantation. Primary prevention of sudden cardiac death was more common in the LBBB group. Other indications, including second-degree atrioventricular block, sinus node dysfunction, and atrial tachyarrhythmias with bradycardia, were infrequent reasons for PPM in either group. In a multivariate linear regression model, chronic lung disease (estimate = 0.67; 95% CI: 0.03–1.32, *p*-value = 0.04) and higher Society of Thoracic Surgeons (STS) risk score (estimate = 0.13; 95% CI: 0.05–0.22, *p*-value = 0.003) were associated with longer hospitalizations. Patients who required a pacemaker during their hospital stay had an average increase in length of stay by approximately 1.85 days compared to those who did not (estimate = 1.85; 95% CI: 0.93–2.78, *p*-value = 0.0001, Supplementary Table 1). The model was also adjusted for type of baseline BBB, which was not significant. Other factors, such as age, atrial fibrillation (AF) history, hypertension, and diabetes, showed trends but were not statistically significant.

### Predictors of Need for PPM

In multivariable logistic regression analysis evaluating predictors of early PPM implantation within 30 days after TAVR (Table 3), a history of AF or flutter and right-sided ID (MSID) were independently associated with PPM requirement. Patients with a history of AF or flutter had significantly higher odds of requiring PPM implantation compared with those without (odds ratio [OR] = 4.13; 95% CI: 1.63–10.45; *p*-value = 0.003). In contrast, deeper right-sided ID was inversely associated with PPM implantation (OR = 0.90; 95% CI: 0.81–0.99; *p*-value = 0.04).

**Table 3:** Predictors of permanent pacemaker implantation at 30 days after TAVR in patients with BBB

Variable	Odds ratio	95% CI	*p*-value
History of atrial fibrillation/flutter	4.13	1.63–10.45	**0.003**
MSID right-side	0.90	0.81–0.99	**0.04**
Pre-TAVR LVEF (%)	0.97	0.93–1.01	0.14
Total annular calcium volume	1.00	1.00–1.00	0.38
Baseline RBBB (vs. LBBB reference)	3.56	1.01–12.58	**0.04**
Predilation	1.54	0.51–4.65	0.44
Preprocedure creatinine ≥ 2 mg/dL	2.34	0.63–8.73	0.21
Pre-TAVR PR interval (ms)	1.00	0.99–1.01	0.80
TAVR access site: nonfemoral	0.29	0.03–2.88	0.29
Valve size >23 mm	0.62	0.23–1.68	0.34

*Notes*. Significant *p*-values (<0.05) are highlighted in bold.

Abbreviations: BBB, bundle branch block; LBBB, left bundle branch block; LVEF, left ventricular ejection fraction; MSID, membranous septum implantation depth; RBBB, right bundle branch block; TAVR, transcatheter aortic valve replacement.

Baseline conduction subtype was also included in the model, with LBBB used as the reference category. In this early PPM model, baseline RBBB was independently associated with an increased likelihood of PPM implantation (OR = 3.56; 95% CI: 1.01–12.58; *p*-value = 0.04).

In a secondary multivariable logistic regression model assessing cumulative PPM implantation over the entire follow-up period (up to 2 years) (Supplementary Table 3), baseline conduction subtype was not independently associated with PPM implantation (RBBB vs. LBBB reference: OR = 1.92; 95% CI: 0.75–4.91; *p*-value = 0.18). In this longer-term model, AF or flutter was the only independent predictor of PPM placement (OR = 3.11; 95% CI: 1.38–7.01; *p*-value = 0.01), whereas right-sided ID, along with other clinical, anatomical, and procedural variables, was not. In addition, history of AF/flutter and right-sided ID were the only independent predictors of PPM placement during index hospitalization (Supplementary Table 2)

## Discussion

The key findings of our study were as follows: (1) PPM implantation was significantly earlier and more frequent in patients with preexisting RBBB compared with LBBB at 2, 3, 7, and 30 days after TAVR; (2) in-hospital PPM placement was associated with increased length of hospital stay; (3) there were no significant differences in all-cause mortality or heart failure hospitalization between RBBB and LBBB patients, irrespective of post-TAVR PPM implantation status at 2-year follow-up; (4) pre-TAVR RBBB and prior AF/flutter were independently associated with increased 30-day PPM risk, whereas deeper right-sided MSID was associated with lower PPM risk; and (5) CHB was the leading PPM indication in RBBB patients, although prevention of sudden cardiac death was more common in LBBB patients.

Among patients with preexisting BBB undergoing TAVR, the cumulative incidence of new PPM is higher in those with RBBB (24.54%) compared to LBBB (17.12%) at 2 years. Preexisting RBBB was associated with significantly higher and earlier rates of PPM implantation after TAVR compared with LBBB, with the greatest divergence occurring within the first week. By 7 days, nearly 17.84% of RBBB patients required PPM versus 6.31% of those with LBBB, and this disparity persisted through 30 days and long-term follow-up. In contrast, these differences in conduction vulnerability did not translate into differences in clinical outcomes: Kaplan–Meier analyses showed no significant differences in all-cause mortality or heart-failure hospitalization between RBBB and LBBB at 2 years. Furthermore, stratifying patients by PPM status did not meaningfully alter survival curves in either conduction-pattern group. Together, these findings highlight that although RBBB patients show higher observed rates of PPM implantation, their long-term outcomes remain comparable to those with LBBB. Our results are consistent with prior literature, which has reported that long-term survival is similar regardless of whether patients receive a PPM after TAVR.^20^ Factors associated with the increased PPM risk at 30 days included pre-procedural AF/flutter, lower ID near the membranous septum, and pre-TAVR RBBB. Predictors of PPM implantation differed according to timing after TAVR. Baseline conduction subtype and procedural factors were associated with PPM implantation within 30 days, whereas no independent association was observed between baseline BBB subtype and cumulative PPM implantation over longer-term follow-up at 2 years. This pattern mirrors the cumulative incidence curves in Figure 2, which demonstrate early separation between RBBB and LBBB patients with no statistical difference in event rates beyond 30 days. CHB is the most common indication for PPM in RBBB patients, whereas PPMs are more often implanted prophylactically to prevent sudden cardiac death in LBBB patients.

The prevalence of RBBB and LBBB was 13.4 and 5.5% in our cohort, respectively. These findings align with previous studies, which have reported RBBB prevalence rates ranging from 6.9 to 13.6%.^21^^,^^22^ Around 10% had baseline LBBB in a recent meta-analysis of 33 studies.^23^ Although the PPM rate observed in our study was elevated, it remained lower than previously reported rates in populations with baseline BBB.^8^^,^^9^^,^^23^ A recent meta-analysis reported the incidence of new PPM to be 19.6% to 30.6% with studies reporting an incidence of up to 40.1% in patients with preexisting RBBB.^23^ This is significantly higher than the incidence observed in the general TAVR population which ranges from 8% to 10% for balloon-expandable valves and 12% to 15% for self-expanding valves.^16^ The high incidence of new PPM in patients with preexisting RBBB may be due to the close anatomical relationship between the AA and the bundle of His.^2^ Since the left bundle branch lies near the annulus, especially close to the noncoronary and right coronary cusps, deep valve implantation can increase the risk of injuring the left bundle and causing CHB.^24^

We found that patients with a history of AF/flutter have more than twice the odds of requiring a pacemaker after TAVR, consistent with a meta-analysis of over 980,000 patients that demonstrated a modest positive association between AF and PPM implantation.^25^ Preexisting AF/flutter may be a marker of advanced atrial conduction system remodeling in older patients with AS, including atrial fibrosis and enlargement. In this context, AF/atrial flutter has been associated with higher rates of PPM implantation after TAVR, likely reflecting an underlying vulnerable conduction substrate.^26^ Each 1 mm increase in MSID reduces the odds of PPM by approximately 9%, underscoring its protective role. MSID offers more clinical insight than MS length alone, as it accounts for both anatomy and ID and allows for deeper valve placement without necessarily increasing conduction risk. A meta-analysis showed that each 1 mm decreased in ΔMSID raises the odds of PPM by 75%.^27^ Similarly, Isogai et al*.*^8^ identified greater ID as an independent predictor of lower HAVB, CHB, and 30-day PPM/ICD rates. Valve size and predilation were not significant predictors of PPM in our cohort, although the limited use of self-expandable valves restricts comparisons. Prior studies suggest certain valves, like Medtronic CoreValve and Evolut Pro, may increase PPM risk in patients with baseline RBBB.^9^

Data on PPM timing and indications in patients with BBB are limited. Among 85 patients who received PPM in our cohort at 2-year follow-up, 51 received in-hospital PPM, and the cumulative number of PPMs within 30 days postdischarge was 58. In-hospital, the majority of PPM was placed on day 1 of hospital stay. These findings are comparable to those reported in the PARTNER trial registry and earlier smaller studies.^28^^,^^29^ The most common indication for new PPM is CHB (58%), followed by primary prevention of sudden cardiac death (14%). As expected, CHB was more frequent among patients with preexisting RBBB, aligning with PARTNER trial data, where HAVB or CHB accounted for 79% of PPM cases, followed by sick sinus syndrome.^28^

## Limitations

This study has several limitations inherent to its retrospective, single-center design, which may restrict the generalizability of the findings. Despite a substantial prevalence of preexisting BBB, the relatively low rate of PPM implantation may reflect center-specific practices or patient selection and may not be representative of broader clinical populations.

The study was also underpowered to detect differences in downstream clinical outcomes such as mortality and heart failure hospitalization. Although prior studies have suggested an association between post-TAVR pacemaker implantation and adverse outcomes, the absence of such associations in the present analysis may represent a false negative finding due to limited event rates. Furthermore, implantation techniques likely evolved over the study period, including trends toward higher or more optimized implantation depths, which may have influenced conduction outcomes and introduced temporal confounding. Finally, the inclusion of patients who underwent pacemaker or ICD implantation for indications unrelated to conduction disturbances, such as primary prevention of sudden cardiac death, may have affected the observed associations.

## Conclusion

This study shows that the incidence of PPM is high in patients with preexisting BBB, but it is not as high as previously reported in the literature. Patients with preexisting RBBB experienced higher and earlier rates of PPM implantation following TAVR compared with those with LBBB, whereas long-term outcomes remained similar regardless of conduction pattern or pacemaker status. Preprocedural AF/flutter and pre-TAVR RBBB were strong predictive factors of PPM placement at 30 days, whereas high valve implantation depth can minimize the risk of PPM even in this high-risk population.

## Ethics Statement

This study was conducted in accordance with the relevant ethical guidelines. The study protocol was reviewed and approved by the institutional review board/ethics committee.

## Review Statement

The review of this manuscript was managed by Guest Editor Dr Jorge Nuche.

## Funding

This study was possible with a generous gift from Jennifer and Robert McNeil. The funders had no rule in any stage of the manuscript.

## Disclosure Statement

The authors report no conflict of interest.
