# Supplementary material for: Pacemaker Implantation in Patients With Pre-Existing Bundle Branch Block Undergoing Balloon-Expandable Transcatheter Aortic Valve Replacement
Source: Struct Heart. 2026 May 22;10(8):101052. doi: 10.1016/j.shj.2026.101052 (PMC13449774; doi:10.1016/j.shj.2026.101052)
Supplement: Supplementary file 1 [file mmc1.docx]

**Supplementary Material**

**Supplementary Table 1: Multivariate Linear Regression Analysis of Factors Associated with Length of Hospitalization post-TAVR**

| **Variable** | **Β Estimate** | **95% CI** | **P-value** |
| --- | --- | --- | --- |
| Age (per year) | -0.02 | -0.06 to 0.02 | 0.29 |
| Female sex | 0.14 | -0.55 to 0.84 | 0.69 |
| Coronary Artery Disease | 0.52 | -0.18 to 1.22 | 0.15 |
| Prior Stroke | 0.67 | -0.20 to 1.54 | 0.13 |
| Hypertension | 0.11 | -0.92 to 1.15 | 0.83 |
| Diabetes | 0.10 | -0.53 to 0.73 | 0.76 |
| Chronic Lung Disease | 0.67 | 0.03 to 1.32 | **0.04** |
| History Atrial Fibrillation/flutter | 0.29 | -0.39 to 0.97 | 0.40 |
| STS Risk Score | 0.13 | 0.05 to 0.22 | **0.003** |
| BMI (Kg/m^2^) | 0.04 | -0.01 to 0.08 | 0.08 |
| Pre-procedural Creatinine ≥ 2 mg/dL | 0.53 | -0.59 to 1.65 | 0.35 |
| Urgent/Emergency Status | 0.94 | -0.11 to 1.99 | 0.08 |
| Pre-TAVR LVEF (%) | -0.01 | -0.03 to 0.02 | 0.64 |
| Prior Myocardial Infarction | -0.33 | -1.15 to 0.49 | 0.43 |
| General Anesthesia (vs Moderate Sedation) | -0.05 | -0.94 to 0.85 | 0.92 |
| Perforation±Tamponade | 1.40 | -1.58 to 4.37 | 0.36 |
| In-Hospital Ischemic Stroke | -0.49 | -5.63 to 4.64 | 0.85 |
| New-onset AF In-Hospital | -0.46 | -2.45 to 1.53 | 0.65 |
| Baseline RBBB (vs LBBB reference) | -0.02 | -0.75 to 0.70 | 0.95 |
| Procedural Complete Heart Block | 0.47 | -0.56 to 1.49 | 0.37 |
| HR at Wenckebach Onset | -0.46 | -1.17 to 0.25 | 0.20 |
| Pre-TAVR Ventricular Rate | 0.01 | -0.01 to 0.03 | 0.38 |
| Pre-TAVR PR Interval (ms) | -0.00 | -0.01 to 0.00 | 0.38 |
| Pre-TAVR QRS Duration (ms) | 0.01 | -0.02 to 0.03 | 0.59 |
| Aortic Valve Mean Gradient (mmHg) | 0.01 | -0.01 to 0.03 | 0.49 |
| White Race (vs Other) | -0.30 | -1.66 to 1.07 | 0.67 |
| In-Hospital permanent pacemaker implantation | 1.85 | 0.93 to 2.78 | **0.0001** |

**Supplementary Table 2: Predictors of In-Hospital Permanent Pacemaker Implantation in post-TAVR Patients with BBB.**

| **Variable** | **Odds Ratio** | **95% CI** | **p-value** |
| --- | --- | --- | --- |
| History of Atrial fibrillation/flutter | 4.44 | 1.55 – 12.71 | **0.01** |
| MSID right side | 0.83 | 0.73 – 0.95 | **0.01** |
| Pre-TAVR LVEF | 0.98 | 0.94 – 1.03 | 0.46 |
| Total Annular Calcium volume | 1.00 | 1.00 – 1.00 | 0.31 |
| Baseline RBBB (vs LBBB as a reference) | 3.67 | 0.85 – 16.00 | 0.08 |
| Predilation | 2.09 | 0.65 – 6.76 | 0.22 |
| Pre-procedural Creatinine ≥ 2mg/dL | 2.79 | 0.69 – 11.34 | 0.15 |
| Pre-TAVR PR interval (ms) | 1.01 | 0.99 – 1.02 | 0.29 |
| TVT Access Site: Non-femoral | 0.35 | 0.03 – 3.67 | 0.38 |
| Valve size >23 mm | 0.62 | 0.20 – 1.92 | 0.40 |

**Supplementary Table 3: Predictors of Permanent Pacemaker Implantation at 2 years post-TAVR in Patients with BBB.**

| **Variable** | **Odds Ratio** | **95% CI** | **p-value** |
| --- | --- | --- | --- |
| History of Atrial fibrillation/flutter | 3.11 | 1.38 – 7.01 | **0.01** |
| MSID right-side | 0.92 | 0.85 – 1.01 | 0.08 |
| Pre-TAVR LVEF (%) | 0.99 | 0.95 – 1.03 | 0.55 |
| Total Annular Calcium volume | 1.00 | 1.00 – 1.00 | 0.42 |
| Baseline RBBB (vs LBBB as a reference) | 1.92 | 0.75 – 4.92 | 0.18 |
| Predilation | 1.15 | 0.43 – 3.09 | 0.78 |
| Pre-procedural Creatinine ≥ 2mg/dL | 2.15 | 0.64 – 7.19 | 0.22 |
| Pre-TAVR PR interval (ms) | 1.00 | 0.99 – 1.01 | 0.51 |
| TVT Access Site: Non-femoral | 0.23 | 0.03 – 2.11 | 0.19 |
| Valve size >23 mm | 0.59 | 0.25 – 1.41 | 0.24 |
